# Reported circulation of Zika and re-circulation of Chikungunya in Dengue-endemic Dhaka, Bangladesh in 2024

**DOI:** 10.1371/journal.pone.0340119

**Published:** 2026-02-12

**Authors:** Rahman Mizanur, Khan Fariduddin Ayaz, Hasan Nazmul, Rummana Rahim, Abu Hasan, Naoko Kawai, Tatsuki Sugi, Kyoko Hayashida, Junya Yamagishi

**Affiliations:** 1 Molecular Diagnostics, Evercare Hospital Dhaka, Dhaka, Bangladesh; 2 Department of Medicine, Evercare Hospital Dhaka, Dhaka, Bangladesh; 3 International Institute for Zoonosis Control, Hokkaido University, Sapporo, Japan; CEA, FRANCE

## Abstract

**Background:**

In Dhaka, Bangladesh, dengue has been endemic for more than two decades. With the explosive outbreak of Chikungunya in 2017 and the recent report of Zika in 2023, we started routine screening of Dengue virus (DENV), Chikungunya virus (CHIKV) and Zika virus (ZIKV) of febrile patients attended in our tertiary care hospital to overcome diagnostic challenges posed by the symptomatic similarities of these viral infections.

**Method:**

Serum samples of 603 febrile patients from August to December 2024 were tested by multiplex RT-PCR to detect DENV, CHIKV and ZIKV. Genome sequencing and phylogenetic analysis were performed for some of these samples.

**Result:**

A total of 300 (49.8%) patients were positive for one or more viruses: 85 (14.1%) for DENV, 188 (31.18%) for CHIKV and 33 (5.47%) for ZIKV. Four CHIKV-DENV and two CHIKV-ZIKV co-infections were found. CHIKV sequences obtained in this study belonged to the ECSA clade and highly like those circulated in India from 2018 to 2024 but distinguished from Bangladesh strain of 2017. On the other hand, Bangladesh ZIKV sequences in 2024 belonged to the Asian clade and Southeast Asian subtype. They were most similar to strains circulated in Bangladesh in 2023, followed by sequences from Cambodia (2019), Thailand (2016) and Singapore (2016).

**Conclusion:**

Thus, these findings underscore the need for enhanced surveillance and public health interventions to mitigate the spread and impact of these viral infections in arboviral-endemic regions.

## Introduction

The Asia Pacific region is highly vulnerable to the effects of climate change and outbreaks of climate-sensitive viral infectious diseases, such as dengue, chikungunya, and Zika. These viruses are transmitted by *Aedes* mosquitoes which are available ubiquitously in Dhaka, Bangladesh (https://www.bmrcbd.org/Bulletin/bulletin_html/4502_Editorial.html) and the population of Aedes mosquitoes in Dhaka’s two city corporations has almost tripled in the last four years, according to a monsoon survey report 2023 from the Directorate General of Health Services (DGHS) (https://www.tbsnews.net/tags/aedes-mosquito). These three are single-stranded, positive-sense RNA viruses, of which dengue virus (DENV) and Zika virus (ZIKV) belong to the genus *Orthoflavivirus* in the family *Flaviviridae*, whereas chikungunya virus (CHIKV) is a member of the *Alphavirus* genus in the family *Togaviridae* (https://ictv.global/taxonomy). The clinical manifestations of these viruses are diverse, ranging from asymptomatic infection to mild and self-limited febrile illness, in most cases. However, long term chronic joint pain (arthritis) in some cases in chikungunya, congenital anomalies in Zika and fatal outcome in dengue are also reported [1,2]. Currently, there are no specific treatments available for these arboviral infections (https://health.ny.gov/diseases/communicable/arboviral/fact_sheet.htm). Recently, one FDA approved vaccine is available in USA for chikungunya virus, and vaccine for Zika virus is still under development [3].

Dengue has been endemic in Bangladesh since 2000 [4]. On the other hand, the emergence of Zika in Bangladesh was first reported by a single case in 2014 [5] and then five more cases in 2023 including one DENV-ZIKV co-infection [6]. Zika virus is an emerging and re-emerging virus of medical and public health importance [7]. The World Health Organization (WHO) has already declared ZIKV infection a new emerging disease to be managed [8]. At present this infection is seen worldwide in many countries, including our neighbor country India, Myanmar and Thailand [9–11]. Besides, chikungunya was first reported in 2008 in Bangladesh followed by a massive outbreak observed in 2017 [12] and thereafter, no cases had been reported in the past several years. Clinical suspicion of chikungunya again appeared from middle of 2024. As there was recent report of Zika in dengue endemic Dhaka and as symptoms overlap in these arboviral infections, confirmation of diagnosis without laboratory evidence was difficult. To detect and differentiate DENV, CHIKV and ZIKV, we routinely started screening of febrile patients by multiplex RT-PCR from August 2024. Furthermore, we conducted partial and whole genome analyses of CHIKV and ZIKV circulating in Dhaka in 2024, respectively, to elucidate their genetic diversity, estimate transmission pathways, identify key mutations, and trace evolutionary trajectories that may inform disease control efforts.

## Materials and methods

### Ethical approval

This study was approved by the Research and Ethical Practice Committee of Evercare Hospital Dhaka (Approval number: ERC 67/2025-05) on March 5, 2025.

### Method of data collection

Patients with clinical suspicion of dengue, chikungunya or Zika who visited Evercare Hospital Dhaka from August to December 2024 were included in this study. Febrile patients those had suspicion of malaria or bacterial infection were not included. Age, sex, specimen types and clinical history available in hospital information system were used after March 5, 2025 for research purposes. To protect patients’ private information except for age and sex, all samples were de-identified through the Evercare Hospital Information System. De-anonymization was not allowed for research purpose. Patient consent was not needed as data were extracted from the routine test result available in the hospital information system.

### Clinical Samples

Febrile patients who had suspicion of dengue, chikungunya, or Zika underwent routine molecular test for confirmation. These are consecutive hospital laboratory cases. A total of 603 samples from febrile patients were collected from individuals aged 2–89 years at Evercare Hospital Dhaka. For routine assay, 3 ml venous blood sample from adults and 1.5 to 2 ml from pediatric patients having clinical suspicion of dengue, chikungunya, Zika were collected in serum separator tube (SST). Serum was separated and stocked at −80°C until RNA was extracted.

### Cartridge based multiplex PCR by using STANDARD M10

Out of the 603 samples, 549 were tested by the PCR based STANDARD M10 Arbo Panel Cartridge (SD BIOSENSOR, Korea, M10-AB5–01) as per the manufacturer’s instructions at Evercare Hospital Dhaka. Total 600 µl samples were loaded manually into the single-use STANDARD M10 Arbovirus Panel Cartridge. The STANDARD M10 Arbovirus Panel cartridge and STANDARD M10 Analyzer form a closed, fully automated assay. STANDARD M10 system automates and integrates sample preparation, nucleic acid extraction and amplification, and detection of the target sequences in various specimens using molecular diagnostic assays. It is a CE-IVD marked PCR system performs 40 cycles for target amplification. The presence of RNA for DENV, ZIKV and CHIKV in human serum specimen was determined, and results are displayed. The cartridge has in-built positive and negative control, and the validity of each run is determined by amplification of positive control and no amplification of negative control*..*

### RNA Extraction and Real-Time Reverse Transcriptase multiplex PCR

Among 603 samples, 54 were tested by using the Real-Time Reverse Transcriptase multiplex PCR. The RNA was isolated by using FAVORGEN (FavorPrep Viral DNA/RNA kit, Taiwan) spin column-based extraction kit according to the manufacturer’s instructions. A total of 140 µl of sample was used for RNA extraction. The elution volume was 50 µl. We used CE-IVD approved VIASURE ZIKA, DENGUE & CHIKUNGUNYA commercial multiplex real time PCR kit from CerTest Biotec, S.L. Spain for detection of DENV, ZIKV and CHIKV. The total PCR volume was 20 µl where 15 µl rehydration buffer dispenses in lyophilized master mix containing 0.2 ml PCR strip tube for each sample, Negative control (NC) and Positive control (PC). Then 5 µl of extracted RNA, negative control and positive control were added respectively. PCR amplification was done by Quantstudio-5Dx (Applied Biosystems) thermocycler according to kit manufacturer’s instruction. The amplification protocol consisted of 45°C for 15 min followed by 95 °C for 2 min, and then 45 cycles of 95 °C for 10 seconds, and finally 60 °C for 50 seconds. Signals were acquired at 60 °C and analysis was performed using a linear scale. Threshold cycle was determined automatically on most of the runs and occasionally, manually, when more noises are found. Thresholds cycle is not fixed and depends on each run. For the target, any exponential curve crossing this threshold was considered positive. The fluorescence was detected in FAM channel for Dengue, ROX channel for Chikungunya, CY5 channel for Zika and HEX channel for internal control (IC). The recommendations of the manufacturer were strictly followed for RNA extraction and for Real Time PCR. The validity of each run is determined by amplification of positive control and no amplification of negative control.

### CHIKV partial genome sequencing

Partial genome amplification was conducted using a previously reported primer set with index (S1 Table) [12] with minor modifications regarding polymerase and PCR system. Eight randomly selected purified RNA samples were transferred to Japan with dry ice. Obtained amplicons were pooled and purified with NucleoSpin Gel and PCR Clean-up kit (Takara). Sequencing library was constructed using Native Barcoding Kit 96 V14 (SQK-NBD114.96, Oxford nanopore technologies) and sequenced using a FLO-FLG114 flow cell as per the instruction manual. Basecalling and debarcoding were performed using dorado (Oxford nanopore technologies), producing pooled CHIKV FASTQ sequences from the eight samples, each uniquely indexed by sequences at both ends [12]. Excess adapter sequences were trimmed with porechop [13]. The index sequence were identified using minibar [14]. Sequences with more than two mismatches in each index sequences were subtracted. Index and primer sequences were trimmed using minibar as well. Consensus sequences of each sample were constructed using amplicon sorter [15] with the following options: -min 900 -max 1100 -sg 80 -sc 95.

### ZIKV whole genome sequencing

The whole genome amplification was conducted in Japan using previously reported 11 primer sets [16], covering 98.6% of the whole genome excluding both ends, with minor modifications regarding polymerase and PCR system. Purified RNA samples with low Ct value (CD-193) and one of the co-infected samples (CD-266) were subjected. Obtained amplicons were pooled and purified with NucleoSpin Gel and PCR Clean-up kit (Takara). The sequencing library was constructed using Native Barcoding Kit 96 V14 (SQK-NBD114.96, Oxford nanopore technologies) and sequenced using a FLO-FLG114 flow cell as per the instruction manual. Basecalling and debarcoding were performed using dorado. Excess adapter and primer sequences were trimmed with porechop [13] and minibar [14], respectively. Alignments were performed using Minimap2 [17] with a reference ZIKV whole genome sequence (NC_035889.1) with -x map-ont option. Primer sequences were trimmed using iVar [18] with -q 3 -m 700 -s 4 option. Consensus sequences were constructed using Medaka (Oxford nanopore technologies) with -M 40 option. The region other than the tiling PCR target were masked by “N”. Partial env gene around the presumable glycosylation site was amplified using OneTaq One-Step RT-PCR Kit (NEB) and the sequences were confirmed by Sanger sequencing.

### Genome Analysis of CHIKV and ZIKV

The obtained partial genome sequences corresponding to 10,204 bp to 11,100 bp of CHIKV genome were aligned with 22 highly similar sequences retrieved from NCBI core nt dataset, each showing over 99.5% sequence similarity as determined by blastn, seven sequences circulated in Bangladesh in 2017 [12] along with five related ones [19] and 35 randomly selected whole CHIKV genomes retrieved from NCBI using mafft [20]. After removing gaps, 897-bp aligned sequences were used to construct a phylogenetic tree by maximum likelihood in MEGA-X [21], employing Semliki forest virus genome (NC_003215.1) as the out group. The analysis was performed with 1,000 bootstrap replicates under the GTR + G + I model selected based on the minimum AICc value.

The obtained whole genome sequences of ZIKV, 15 highly similar sequences (> 98.9%) found in NCBI core nt data set including those circulated in Bangladesh in 2023 and representative sequences of each genotype [22] were retrieved. Their complete poly protein sequences were translated. They were aligned using mafft and gaps were removed. Obtained alignment with 3,423 amino acid sequences each were subjected to describe phylogenetic tree using maximum likelihood in MEGA-X, employing Spondweni virus (NC_029055.1) as the out group. The analysis was performed with 1,000 bootstrap replicates under the JTT + G + F model selected based on the minimum AICc value. Criteria for subtypes followed a previous study [22]. Furthermore, we thoroughly compared the distinctive deletion sequences observed in the Bangladesh 2023 genomes [6]. The sequences corresponding to genome positions 1388–1489 nt were retrieved from three Bangladesh 2023 genomes, two Bangladesh 2024 genomes in this study, three additional Bangladesh 2024 sequences amplified by specific RT-PCR, six genomes selected from the phylogenetic analysis, and two representative reference genomes (NC_012532, MR 766 strain; KF268948, ARB13565 isolate). The translated amino acid sequences were aligned using MAFFT [20], and the corresponding nucleotide sequences were aligned according to this amino acid alignment.

## Results

### Prevalence and clinical manifestations

**Fig 1:**
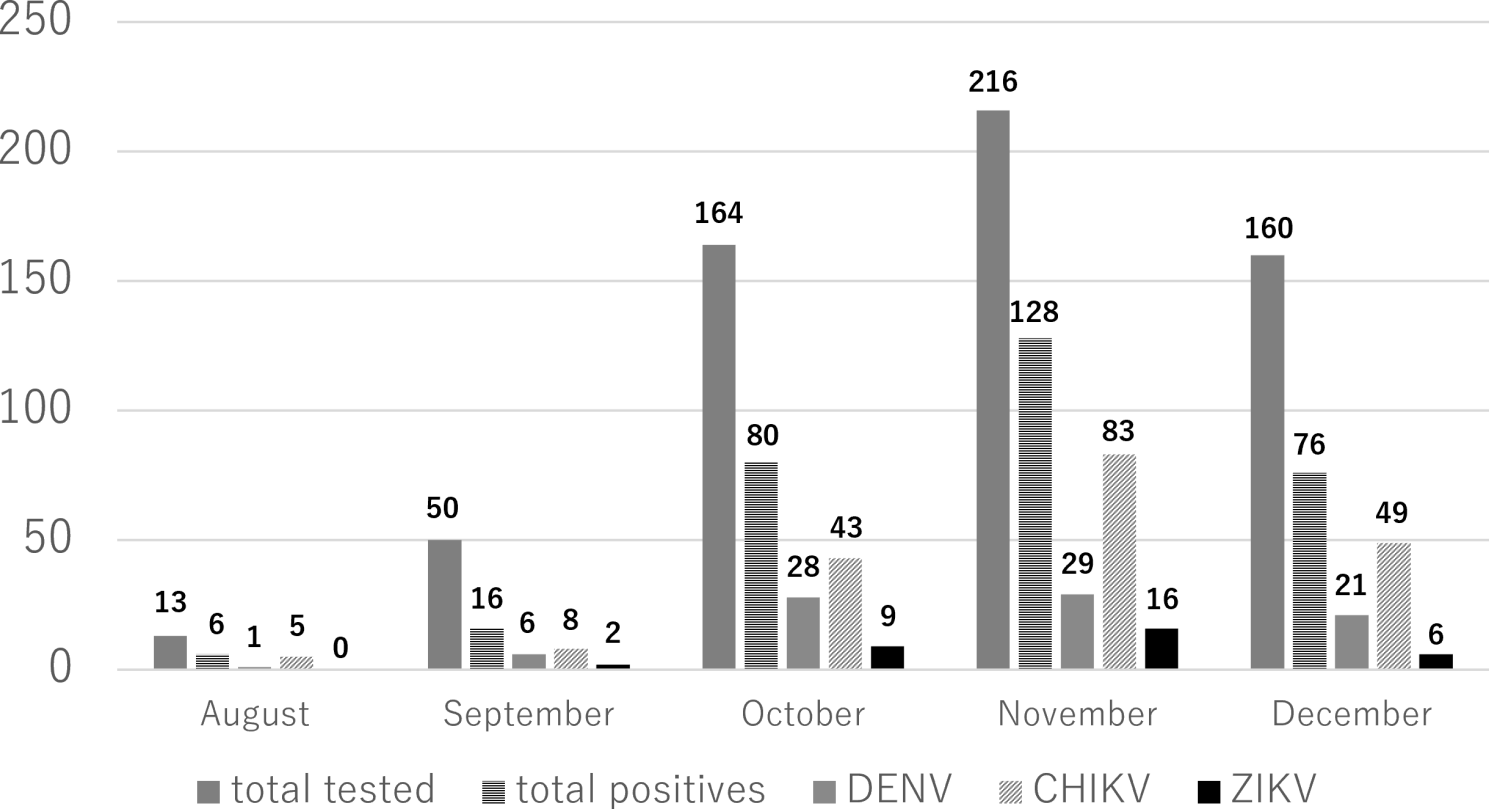
Distribution of cases and confirmed number of DENV, CHIKV and ZIKV during August to December 2024 in Bangladesh.

The age of the patients ranged from 2 to 89 years. Out of the total tested cases, 288 were male and 315 were female. Among the 603 samples tested, 300 (49.8% ± 4.0%) were positive for at least one. During this five-month period, the overall suspected cases and positivity rate gradually increased and reached highest in the month of November and then decreased (Fig 1). Of these 300 positive cases, 85 (14.1%) were DENV, 188 (31.2%) were CHIKV and 33 (5.5%) were ZIKV positive (S2A-C Table). Among them, two cases were co-infected with CHIKV and ZIKV, while four were co-infected with CHIKV and DENV. There were no triple co-infections.

Out of the 188 CHIKV positive patients, 93 patients were male and 95 were female. Among them, 124 patients were treated as outpatient and the remaining 64 were admitted to the hospital. Clinical presentation data were available for 169 patients (Table 1). Among them, all the patients were febrile. The other major clinical manifestations were myalgia (80.5%) followed by arthralgia (42.6%), rash (18.9%), headache (11.2%), respiratory tract infection (4.7%) and conjunctivitis (0.6%). Myalgia and arthralgia were higher in female patients than in male patients. We found hematological parameters of 175 patients. Among them, the most common hematological abnormalities were mild to moderate anemia (58.3%) and lymphopenia (45.1%). Other variables were leukopenia (23.4%), neutropenia (11.4%) neutrophilia (9.1%), thrombocytopenia (3.4%), leukocytosis (2.9%), lymphocytosis (0.6%).

**Table 1:** Clinical and Hematological profiles of the study population.

	Zika			Chikungunya		
Clinical Manifestations	male	female	total	male	female	total
Total case	12	21	33	86	83	169
Fever	12	21	33	86	83	169
Rash	4^*^	19	23	11	21	32
Headache	3	2	5	11	8	19
Myalgia	9	10	19	66	70	136
Arthralgia	5	12	17	35	37	72
Conjunctivitis	2	6	8	1	0	1
Upper respiratory tract infection	3	4	7	5	3	8
Hematological Profile	male	female	total	male	female	total
total case	12	20	32	90	85	175
Anemia	3	5	8	50	52	102
Leukocytosis	1	0	1	1	4	5
Leukopenia	1	1	2	23	18	41
Neutrophilia	1	1	2	8	8	16
Neutropenia	1	1	2	10	10	20
Lymphocytosis	0	1	1	0	1	1
Lymphopenia	1	5	6	43	36	79
Thrombocytopenia	2	1	3	4	2	6

*) Significant (p < 0.05, binomial test with Bonferroni correction)

Among the 33 ZIKV positive patients, 12 patients were male and 21 were female (Table 1). Of these positive cases 27 patients were treated as outpatient, while the remaining 6 were admitted. Fever was reported by all patients. The other major clinical presentation was rash (69.7%), followed by myalgia (57.6%), arthralgia (51.5%), conjunctivitis (24.2%), upper respiratory tract infection (21.2%) and headache (15.2%). Rash was higher in female patients than in male patients with statistical significance (p < 0.05) for reasons unknown. Hematological data was found for 32 patients. Among them common variations in hematological parameters were mild to moderate anemia (25.0%) and lymphopenia (18.8%). Other variables were thrombocytopenia (9.4%), leukopenia (6.3%), neutropenia (6.3%), neutrophilia (6.3%), lymphocytosis (3.1%).

### CHIKV genome analysis

**Fig 2:**
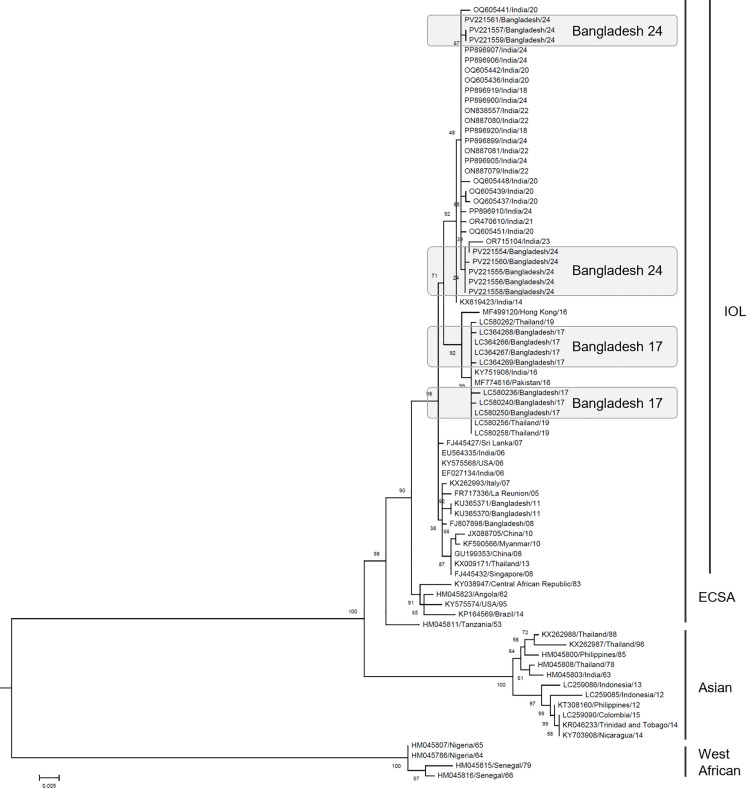
Phylogenetic tree of Chikungunya virus based on nucleic acid sequences. ECSA and IOL stand for East-Central-South-African and Indian Ocean Lineage, respectively. Bootstrap value was added around each blanch. Each sequence was labeled as genbank ID/isolated country/isolated year. The list was provided as S4A Table. Sequences originated from Bangladesh in 2017 and 2024 (this study) were emphasized by gray box.

With Nanopore multiplexing sequencing and demultiplexing, we obtained 154–831 reads for the eight samples. Each consensus sequence of 973 bp was obtained using these reads. They were supported by more than 150 reads. The most similar sequences in NCBI core nt dataset were retrieved and most of them were CHIKV circulated in India from 2018 to 2024. These sequences were aligned together with partial genome sequences of CHIKV circulated in Bangladesh in 2017 and representative CHIKV genome sequences then a phylogenetic tree was described (Fig 2). Firstly, the eight CHIKV sequences obtained in this study belonged to the ECSA clade and highly similar to those circulated in India from 2018 to 2024 (Fig 2). The Bangladesh 2024 sequences appeared to form two groups, but it was not obvious considering the bootstrap values. On the other hand, the Bangladesh 2024 were distinguished from the Bangladesh 2017 (Fig 2).

One of the cluster including PV221557 and PV221559 shared a C10907T substitution in the reference genome NC_004162.2, corresponding to an A1114V amino acid change in the structural polyprotein (NP_690589.2). Using InterPro, it was predicted that the substitution was corresponding to E set domain in the E1 glycoprotein. However, the impact of the mutation on the viral function remains unknown. The other cluster including PV221554, PV221560, PV221555, PV221556 and PV221558 shared C10371T substitution but it was synonymous.

### ZIKV genome analysis

**Fig 3:**
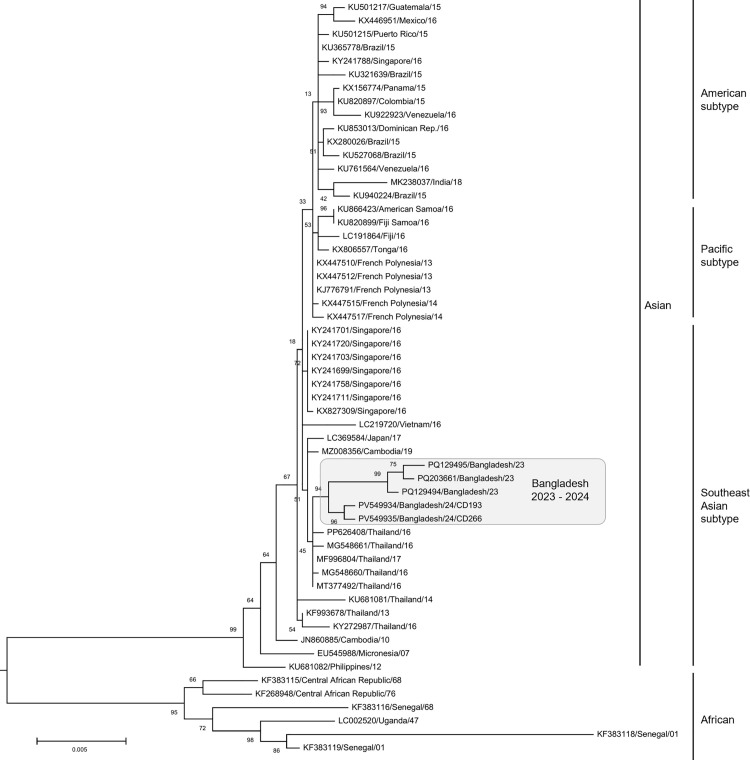
Phylogenetic tree of Zika virus based on amino acid sequences. Bootstrap value was added around each blanch. Each sequence was labeled as genbank ID/isolated country/isolated year. The list was provided as S4B Table. Sequences originated from Bangladesh in 2023 and 2024 (this study) were emphasized by gray box.

To obtain whole genome sequences of ZIKV circulated in Bangladesh in 2024, one clinical sample co-infected with CHIKV (CD266) and one clinical sample with relatively high Ct value (CD193) were selected. The genome was amplified with tiling PCR of 11 fragments as per the published method [16] with some modifications. Using the re-sequencing approach, a total of 40,098 and 150,297 reads were aligned to the reference genome for CD-193 and CD-266, respectively (S3 Table), then 10,660 bp nearly complete genome sequences with complete CDS were obtained for the both. They were most similar to Bangladesh 2024 followed by those circulated in Thailand and Singapore in 2016. No sequences were available to bridge the temporal gap between 2016 and 2023. Their amino acid sequences based phylogenetic tree demonstrated that the Bangladesh sequences in 2024 belonged to the Asian clade and Southeast Asian subtype together with those in 2023 (Fig 3).

**Fig 4:**
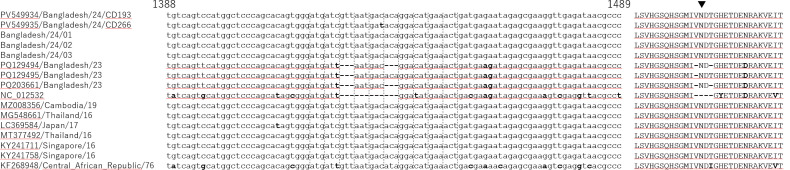
Alignment of the ZIKV envelope protein region with deletions. The left represent nucleotide alignment from 1388 bp to 1489 bp. The dashed lines represent codon flame. The right represent corresponding amino acid alignment. The hyphens represent deletion. Mismatches were shown in bold. Predicted N glycosylation sites were shown by the arrowhead.

Finding common substitutions which differentiate the Bangladesh genomes from others, the 15 highly similar ones, PQ129494.1, MZ008356.1, PQ129495.1, PQ203661.1, MF996804.1, PP626408.1, MG548660.1, MG548661.1, LC369584.1, MT377492.1, KY241711.1, KY241758.1, KY241699.1, KY241703.1 and KY241701.1 were aligned with the Bangladesh 2023–2024 genomes. As a result, 38 Bangladesh 2023–2024 specific, 20 Bangladesh 2023 specific and three Bangladesh 2024 specific substitution were identified (Table 2). Among them, there were six non-synonymous mutations. Their potential biological functions were examined using InterPro; however, the limited information did not allow us to propose a reliable hypothesis. In addition, there were four deletions and one insertion. Among them, three consecutive deletions led to the deletion of one amino acid. The remaining one deletion and one insertion were located in close proximity. This caused a short frameshift, altering their coding amino acid sequences from RREEETP to GGRKRLL. These seven amino acids located between Trypsin-like serine protease and helicase domains of the nonstructural protein NS3; therefore, the impact of substitution might be limited. Furthermore, the distinctive deletion sequences observed in the Bangladesh 2023 genomes [6] were absent in the 2024 genomes (Fig 4).

**Table 2:** Substitutions specific for ZIKV Bangladesh 2023-2024.

Nucleotide^*1^	Amino acid	protein	InterPro	Specificity^*3^
A429G	T108A	anchored capsid protein C		BD23–24
C449T	s. mut.	anchored capsid protein C		BD23–24
T506C	s. mut.	membrane glycoprotein precursor M		BD23–24
T593C	s. mut.	membrane glycoprotein precursor M		BD23–24
A653G	s. mut.	membrane glycoprotein precursor M		BD23–24
C665T	s. mut.	membrane glycoprotein precursor M		BD23–24
G803A	s. mut.	membrane glycoprotein precursor M		BD23
G815A	s. mut.	membrane glycoprotein precursor M		BD23
G1004A	s. mut.	envelope protein E		BD23–24
C1407T	s. mut.	envelope protein E		BD23
1433_1435del	443del	envelope protein E		BD23
G1463A	s. mut.	envelope protein E		BD23
A1464G	N453D	envelope protein E		BD23
C1607T	s. mut.	envelope protein E		BD23–24
C1655T	s. mut.	envelope protein E		BD23–24
C1701T	s. mut.	envelope protein E		BD23–24
C2090T	s. mut.	envelope protein E		BD23–24
C2150T	s. mut.	envelope protein E		BD23
C2294T	s. mut.	envelope protein E		BD23
T2298C	s. mut.	envelope protein E		BD24
C2441T	s. mut.	envelope protein E		BD23–24
A2531G	s. mut.	nonstructural protein NS1		BD23–24
T2555C	s. mut.	nonstructural protein NS1		BD23–24
A2702C	s. mut.	nonstructural protein NS1		BD23–24
C2717T	s. mut.	nonstructural protein NS1		BD23–24
G2837A	s. mut.	nonstructural protein NS1		BD23–24
C2990T	s. mut.	nonstructural protein NS1		BD23–24
G3431A	s. mut.	nonstructural protein NS1		BD23–24
C3594T	s. mut.	nonstructural protein NS2A		BD23–24
T3710C	s. mut.	nonstructural protein NS2A		BD23–24
G3815C	s. mut.	nonstructural protein NS2A		BD23–24
C4086T	s. mut.	nonstructural protein NS2A		BD23–24
T4298C	s. mut.	nonstructural protein NS2B		BD23–24
G4307A	M1400I	nonstructural protein NS2B	Region of a membrane-bound protein predicted to be embedded in the membrane.	BD23–24
C4316T	V1628G	nonstructural protein NS2B	Flavivirus NS3 protease (NS3pro) domain profile.	BD23–24
A4412G	s. mut.	nonstructural protein NS2B		BD23
T4472C	s. mut.	nonstructural protein NS2B		BD23–24
C4623T	s. mut.	nonstructural protein NS3		BD23–24
T4957C	I1617T	nonstructural protein NS3	Flavivirus NS3 protease (NS3pro) domain profile.	BD24
G4988A	s. mut.	nonstructural protein NS3		BD23
T4990G	s. mut.	nonstructural protein NS3		BD23
G4994T	s. mut.	nonstructural protein NS3		BD23
5117delG	*2	nonstructural protein NS3	Flavivirus NS3 protease (NS3pro) domain profile.	BD23
5139_5140insG	*2	nonstructural protein NS3	Flavivirus NS3 protease (NS3pro) domain profile.	BD23
C5357T	s. mut.	nonstructural protein NS3		BD23–24
T5678C	s. mut.	nonstructural protein NS3		BD23–24
T5738C	s. mut.	nonstructural protein NS3		BD23–24
T5783C	s. mut.	nonstructural protein NS3		BD23–24
T6056C	s. mut.	nonstructural protein NS3		BD23
C6645T	s. mut.	nonstructural protein NS4A		BD23–24
C6734T	s. mut.	nonstructural protein NS4A		BD23
A7418G	s. mut.	nonstructural protein NS4B		BD23
A7418G	s. mut.	nonstructural protein NS4B		BD23
A7575T	T2490S	nonstructural protein NS4B		BD24
G7697A	s. mut.	RNA-dependent RNA polymerase NS5		BD23–24
A7749C	K2548Q	RNA-dependent RNA polymerase NS5	capping_2-OMTase_Flaviviridae	BD23
G8096A	s. mut.	RNA-dependent RNA polymerase NS5		BD23–24
A8141G	s. mut.	RNA-dependent RNA polymerase NS5		BD23
A8246G	s. mut.	RNA-dependent RNA polymerase NS5		BD23–24
C9395T	s. mut.	RNA-dependent RNA polymerase NS5		BD23–24
G9884A	s. mut.	RNA-dependent RNA polymerase NS5		BD23–24

*1) corresponding position to a reference genome, Natal RGN strain (NC_035889.1)

*2) amino acids substitution of RREEETP1671_1677GGRKRLL

*3) BD23, BD24 and BD23–24: specific for Bangladesh isolate in 2023, 2024 and both 2023 and 2024, respectively

## Discussion

Dengue fever has been endemic in Bangladesh since 2000 [4]. A single case of Zika virus disease was first reported in 2014 [5], followed by a few cases in 2023 [6], leading to the recent co-circulation of dengue and Zika [6]. Following the Indian Ocean Islands outbreak in 2005–2006 [23], chikungunya fever re-emerged as a massive outbreak in Bangladesh in 2017 [12]. In 2024, circulation was again confirmed, raising concern about a potential future outbreak [24]. Here, we report the continuous co-circulation of dengue and Zika in Bangladesh in 2024, together with the confirmation of chikungunya, resulting in a tripledemic.

We have obtained eight partial genome sequences of CHIKV circulated in Bangladesh in 2024. They belonged to the ECSA genotype Indian Ocean Lineage (IOL) [25] and most similar to those circulated in India from 2018 to 2024 (Fig 2). In contrast, they were distinguished from the clade of Bangladesh in 2017 (Fig 2) suggesting that those of 2024 were not direct offsprings of those of 2017. On the other hand, we have obtained two whole genome sequences of ZIKV circulated in Bangladesh in 2024. They belonged to the Southeast Asian subtype [22] and the most similar to those circulated in Bangladesh in 2023 (Fig 3). However, the ZIKV Bangladesh 2023 had deletions which were absent in those 2024 (Fig 4). When the topology of the phylogeny is also taken into account, it is unlikely that the 2024 ZIKV strains are direct descendants of those from 2023. On the other hand, a group of the second most similar sequences included those identified in Southeast Asia around 2016 (Fig. 3). These findings suggests that the circulating lineage in Bangladesh in 2023 and 2024 has close ancestor; however, it is unclear how the ancestor migrated to Bangladesh, as there are no sequences in the NCBI database more similar than the 2016 viruses that could help bridge the gap.

Interestingly, there were deletions in the envelope protein of ZIKV Bangladesh 2023 (Fig 4). Relevant deletions were sporadically identified regardless of their geographical and temporal factors [26], while it was absent in ZIKV 2024 (Fig 4). Furthermore, asparagine residues in the region are predicted to be glycosylated [27,28]. We examined the effect of the observed deletion in ZIKV Bangladesh 2023 using NetNGlyc [29] then found sequence with one amino acid deletion (PQ129495.1) have more glycosylation potential. In contrast, those with two amino acid deletions (PQ129494.1 and PQ203661.1) lost glycosylation potential, suggesting potential immune evasion associated with the acquired polymorphisms. To obtain further insights, we have sequenced partial genome region around this locus for additional three ZIKV positive samples in 2024 and observed there were no deletions for all of them (Fig 4).

To estimate additional phenotypic differences between ZIKV Bangladesh 2023 and 2024, individual substitutions other than the deletions in the envelope were examined (Table 2). A total of 61 nucleotide substitutions were identified as specific to Bangladesh 2023 and/or 2024, including seven nonsynonymous substitutions and one deletion. Some of these were located within functional regions; however, it remains unclear whether these substitutions lead to functional changes without further molecular virology validation. In addition, a specific set of insertions and deletions in Bangladesh 2023 resulted in a frameshift affecting seven amino acids in the nonstructural protein NS3. It is possible that the consecutive substitutions may also induce functional changes in NS3. To confirm these possibilities, further epidemiological studies and molecular virology validations are required.

Diagnosis of arboviral infection in hospital is conducted based on clinical and laboratory criteria. However, diagnosis based solely on clinical findings is difficult due to the overlapping clinical features caused by these viral infections. Serology and viral RNA detection by PCR is commonly used in clinical laboratories for the diagnosis of arboviral infection. Serology based diagnosis of zika may suffer from misdiagnosis in dengue endemic region due to antibody cross reactions caused by epitope similarities of these flaviviruses. Molecular detection by multiplex RT-PCR is most reliable to diagnose these viruses because of high sensitivity and specificity. Diagnosis of dengue is prioritized in Bangladesh due to huge morbidity and mortality. Chikungunya was prioritized only during the massive 2017 epidemics and thereafter chikungunya was not diagnosed routinely. Truly, sporadic cases are often neglected. We already have detected 188 CHIKV and 33 ZIKV positive cases in this private tertiary care hospital in a season in 2024. So, it is expected that more cases were circulated in the community because only symptomatic patients visit hospital and many people do not produce any symptoms. Further, testing of CHIKV and ZIKV is affected by the number and severity of symptomatic individuals or people suspected of CHIKV or ZIKV infection. Serological test kits and molecular diagnosis can be integrated to detect sporadic and travelers’ infection rapidly and accurately [30,31].

The co-circulation of DENV and ZIKV in Bangladesh began in 2023 [6]. Our study suggests that this co-circulation has continued. Furthermore, the outbreak of CHIKV infection was also confirmed by a previous study [24] and in this study, indicating that a tripledemic occurred in Bangladesh in 2024. In recent years, DENV, ZIKV, and CHIKV infection have also been reported in India, Thailand, and Myanmar [32,33], suggesting a high likelihood of tripledemics in these countries. Similar tripledemics have been documented in the Pacific region during 2012–2014 [22], as well as reported in Brazil and Colombia [20,21].

In 2019 and 2023, large outbreak of dengue was reported to infect about 0.1 million and 0.3 million people in Bangladesh, respectively. However, chikungunya and zika remained under-diagnosed or undiagnosed that misrepresented the actual burden of CHIKV and ZIKV detection. Further, high density of vectors and co-circulation of DENV-CHIKV-ZIKV during the same season are the major concerns. Special attention should be given to diagnose ZIKV infection in pregnant women as having association of fetal microcephaly and neurological complications in ZIKV infection [34,35]. Appropriate etiological diagnosis is needed through combined clinical, epidemiological and laboratory approaches conducted by expert health professionals.

In this study, we demonstrated the co-circulation of DENV, CHIKV, and ZIKV in 2024, Bangladesh. However, as the observation was limited to patients from a single hospital, comprehensive surveillance and integration of epidemiological data are required to understand the nationwide situation in Bangladesh. Furthermore, as the number of genomes obtained in this study was limited, it is difficult to reliably estimate their transmission dynamics, evolutionary trajectories, or identify the genotypes involved in disease manifestation. A larger number of cases is required to reliably characterize the clinical manifestations specific to co-infections as well. Therefore, to advance these studies and mitigate the spread and impact of these viral infections, strengthening surveillance systems, along with further expanding pathogen genome surveillance capacity through collaboration with neighboring countries, is essential.

## Supporting information

S1 Table Primers used in this study. (XLSX)

S2 Table Clinical records of DENV, CHIKV and ZIKV-positive cases. (XLSX)

S3 Table Reads statistics for ZIKV genomes. (XLSX)

S4 Table Genome information used for CHIKV and ZIKV phylogenetic analysis in this study. (XLSX)
